# Eutrophication Reshapes Microbial Communities and Life‐History Strategies in the Riverine Ecosystems

**DOI:** 10.1111/1758-2229.70234

**Published:** 2025-11-21

**Authors:** Haizhou Li, Jing Fu, Xiangyu Fan, Zhiwei He, Yuekai Wang, Shanshan Yang, Jiawang Wu, Li Wu, Jin Zhou

**Affiliations:** ^1^ East China Sea Fisheries Research Institute Chinese Academy of Fishery Sciences Shanghai China; ^2^ State Key Laboratory of Marine Geology Tongji University Shanghai China; ^3^ Department of Molecular and Cellular Biology University of California Davis California USA; ^4^ College of Marine Science and Technology China University of Geosciences Wuhan China; ^5^ School of Marine Sciences Sun Yat‐Sen University Guangzhou China; ^6^ Laboratory for Coastal Ocean Variation and Disaster Prediction, College of Ocean and Meteorology Guangdong Ocean University Zhanjiang China

**Keywords:** ecological model, life‐history strategies, microbiomes, river, urbanisation

## Abstract

Rivers are increasingly affected by human activities, leading to widespread eutrophication. However, the responses of riverine microbiomes to eutrophication remain poorly understood. In this study, we compared microbiomes between eutrophic urban rivers (UR) and relatively undisturbed natural rivers (NR) to elucidate how eutrophication influences community structures, assembly processes, functions and life‐history strategies. Amplicon and metagenomic sequencing revealed that eutrophication substantially enhanced microbial abundance and diversity in riverine ecosystems, with UR harbouring a higher proportion of fast‐growing, nitrogen‐transforming and antibiotic‐resistant taxa. Neutral and null model analyses further revealed that, while stochastic processes predominantly shaped communities in NR, deterministic environmental selection exerted stronger control under eutrophic conditions in UR. Correspondingly, microbial communities in UR exhibited higher 16S rRNA gene copy numbers (median 4.69 vs. 4.28), stronger codon usage bias (0.0209 vs. 0.0204), greater predicted growth rates (0.2664 vs. 0.1567 h^−1^), larger genomes (5.91 vs. 5.19 Mb), higher guanine–cytosine content (57.68% vs. 56.41%) and enriched transposase genes (4.37% vs. 2.98%), collectively indicating a community‐wide shift from *K*‐selected to *r*‐selected life‐history strategies under eutrophication. Overall, this work elucidates how human activities reshape riverine microbial communities and life‐history strategies, providing a basis for predicting the ecological outcomes of nutrient over‐enrichment in fluvial environments.

## Graphical abstract

We use 16S rRNA amplicon and shotgun metagenomic sequencing to assess the microbiome in urban and natural riverine ecosystems. This study reveals that eutrophication reshapes microbial communities and demonstrates a tendency toward *r*‐strategy traits in urban river ecosystems. Urban river microbial life‐history strategy traits are characterised by higher average 16S rRNA gene copy numbers, codon usage bias in ribosomal genes, predicted maximum growth rates, genome sizes, GC content, transposase content and broader niche breadth.

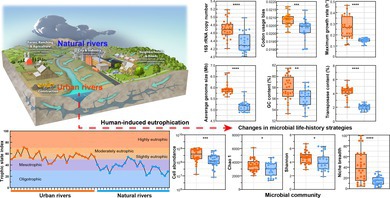

## 1 Introduction

Microbes represent the most metabolically diverse and functionally versatile life forms on Earth (Chen et al. 2021; Osburn et al. 2024). To simplify this complexity, microbial ecologists often classify microbes according to life‐history strategies (Bao et al. 2024; Darling et al. 2012). These strategies comprise sets of life‐history traits that typically co‐vary as a result of physiological or evolutionary trade‐offs, with specific combinations favoured under particular environmental conditions (Li et al. 2025; Yang et al. 2023). Such trait‐based perspectives provide a mechanistic framework linking microbial characteristics to environmental contexts. In microbial ecology, several conceptual models have been proposed to describe these strategies, including the classical *r*/*K* theory, the copiotroph–oligotroph dichotomy, the Yield–Acquisition–Stress tolerance (Y–A–S) framework and the Competitor–Stress tolerator–Ruderal (C–S–R) model (Soler‐Bistué et al. 2023; Couso et al. 2023; Malik et al. 2019).

Among these, the copiotroph–oligotroph dichotomy closely parallels the *r*/*K* classification framework, which differentiates microorganisms by their growth rates, nutrient utilisation strategies, and substrate affinities (Pianka 1970; Yin et al. 2022). This framework has been extensively employed to explain microbial community responses to human disturbances. Copiotrophic, or *r*‐strategist, taxa thrive under nutrient‐enriched conditions, characterised by fast growth rates, low substrate affinities and rapid responses to nutrient inputs (Zhu and Dai 2024). Conversely, oligotrophic, or *K*‐strategist, microorganisms are better adapted to resource‐poor environments, displaying slow growth rates, high substrate affinities and metabolic versatility that enables the degradation of complex or recalcitrant organic matter (Yin et al. 2024; Grime 1977). In essence, the *r*/*K* framework reflects a trade‐off between rapid population expansion and efficient resource utilisation (Ferenci 2016).

The advancement of metagenomic sequencing has enabled exploration of microbial life‐history strategies across diverse ecosystems. A commonly used marker to infer *r*‐ vs. *K*‐strategists is the rRNA operon copy number (Piton et al. 2023; Roller et al. 2016). Species categorised as *r*‐strategists generally possess multiple rRNA operons, facilitating accelerated ribosome synthesis and population expansion under nutrient‐rich conditions (Vieira‐Silva and Rocha 2010). These taxa often display pronounced codon usage bias (CUB) in ribosomal genes, suggesting evolutionary selection toward elevated translational efficiency. Conversely, *K*‐selected microbes usually contain fewer rRNA operons and exhibit more streamlined genomes (Shenhav and Zeevi 2020). Genome streamlining represents an evolutionary adaptation for efficient resource use, allowing oligotrophic taxa to maintain activity and reproduction with minimal energetic expenditure (Giovannoni et al. 2014).

Rivers represent biodiversity hotspots and play central roles in global biogeochemical cycles (Widder et al. 2014; Xie et al. 2016). However, over the past two centuries, intensive industrialisation, urban expansion and agricultural development have substantially increased nutrient inputs to river systems (Beattie et al. 2020; López‐Pacheco et al. 2019). Such nutrient pollution includes organic compounds such as dissolved organic carbon and nitrogen, antibiotics, fertilisers pesticides and animal waste, as well as inorganic nutrients like nitrate and phosphate (Singh et al. 2019; Liu et al. 2023; Pruden et al. 2012; Yang et al. 2022). Excessive nutrient discharge has resulted in nutrient accumulation and frequent eutrophication events (Liu et al. 2021), raising serious concerns about riverine ecosystem health (Zhou et al. 2023; Battin et al. 2016). Despite increasing attention to these issues, it remains uncertain how, and to what extent, anthropogenic eutrophication alters microbial life‐history strategies in riverine environments.

**FIGURE 1:**
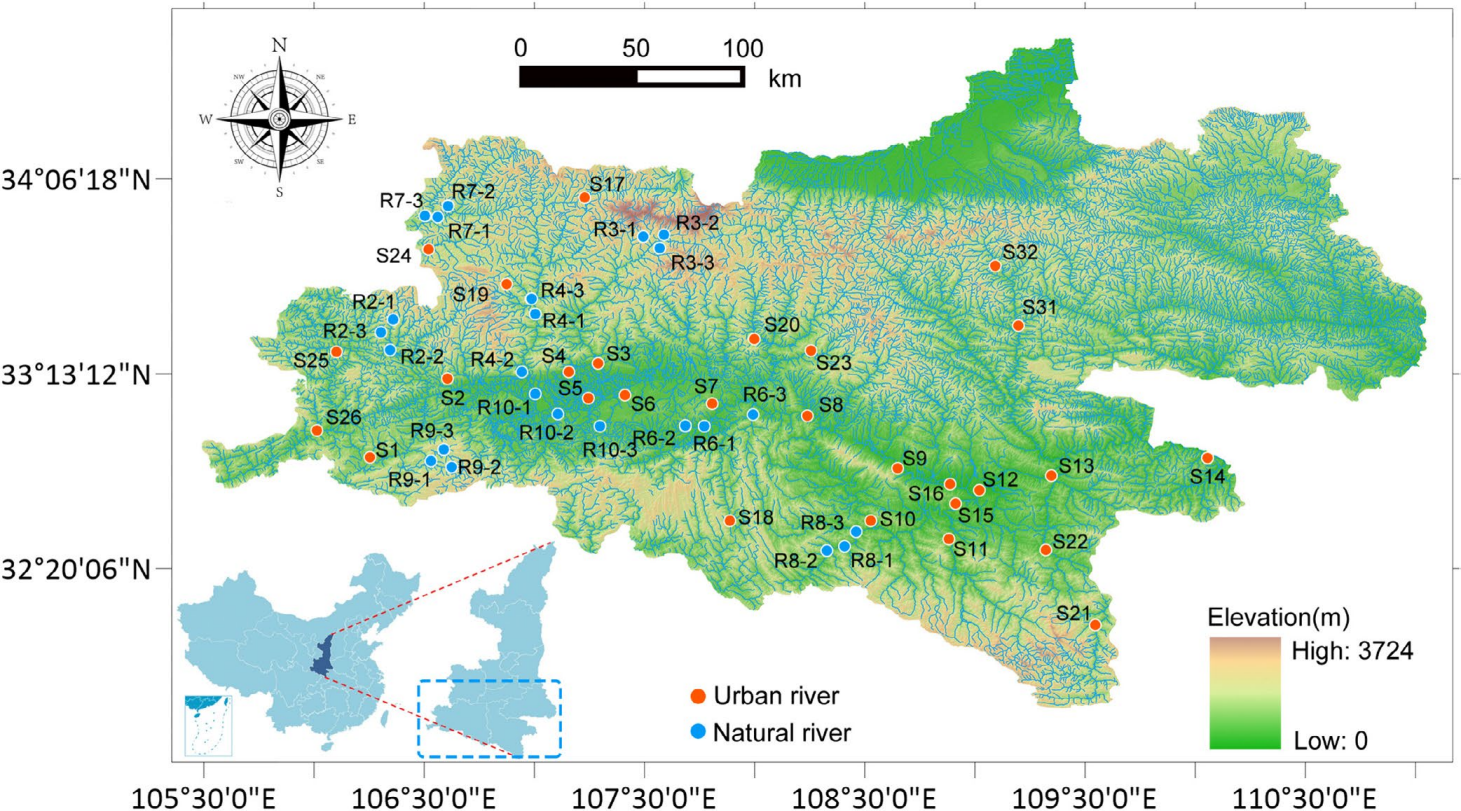
The locations of the river waters and sediments collection sites. The orange circles represent the UR sites, and the blue circles represent the NR sites.

In this study, we focused on a large‐scale river network originating in the Qinling Mountains and flowing into the Yangtze River (Figure 1). The Qinling Mountains, located in northwestern China, form a major north–south climatic boundary and serve as a natural barrier that modulates eastward moisture transport from the Tibetan Plateau, providing freshwater resources for approximately 39 million people (Cheng and Duan 2021). A distinctive feature of this fluvial network is its transition through both densely urbanised and relatively pristine catchments. Leveraging this gradient, sediment samples were collected from highly eutrophic urban rivers (UR) and adjacent, minimally disturbed natural rivers (NR) (Figure 1), enabling a comprehensive assessment of anthropogenic eutrophication effects on the riverine microbiome. By integrating 16S rRNA with metagenomic sequencing, we investigated microbial communities, assembly processes, functions, dissemination of antibiotic‐resistant bacteria (ARB) and life‐history strategies. Our results demonstrate that eutrophication profoundly influences river microbial communities, thereby reshaping microbial life‐history strategies.

## 2 Methods

### 2.1 Physicochemical Analysis

At each sampling location, river water from the upper 50 cm of the water column was obtained using a Schindler sampler, and surface sediments (0–2 cm) were collected with a gravity corer (Figure 1). Geospatial information, including longitude, latitude and intersite distances, was recorded via a GPS unit (Dataset 1A). Water physicochemical variables, such as temperature, pH, dissolved oxygen (DO), Secchi depth (SD), turbidity (TUB), total nitrogen (TN), nitrate‐nitrogen (NO_3_^−^), ammonium‐nitrogen (NH_4_^+^), phosphate (PO_4_^+^), permanganate index, volatile phenol and chlorophyll‐a, were determined in accordance with the Environmental Quality Standards for Surface Waters (GB3838‐2002; Ministry of Ecology and Environment of the People's Republic of China). Sediment total organic carbon (TOC) was quantified using a multi N/C 3100 analyser (Analytik Jena). All analyses were performed in triplicate, and results are reported as mean values (*n* = 3).

### 2.2 Trophic State Index (TSI)

To evaluate the river water trophic status of each site, we calculated a TSI based on the concentrations of TN, TP, Chl‐a and SD in the aquatic environment (Geng et al. 2022; Carlson 1977). TSI was calculated as follows:

(1) $$\begin{array}{cc} \mathrm{TSI}=\; & 0.326^{*}\;\mathrm{TSI}\left(\mathrm{Chl}-a\right)+0.219^{*}\;\mathrm{TSI}\left(\mathrm{TN}\right)\\ & +0.230^{*}\;\mathrm{TSI}\left(\mathrm{TP}\right)+0.225^{*}\;\mathrm{TSI}\left(\mathrm{SD}\right) \end{array}$$

(2) $$\mathrm{TSI}\;\left(\mathrm{Chl}-a\right)=10^{*}\;\left[2.5+1.086^{*}\;\ln\left(\mathrm{Chl}-a\right)\right]$$

(3) $$\mathrm{TSI}\;\left(\mathrm{TP}\right)=10^{*}\;\left[9.436+1.624^{*}\;\ln\left(\mathrm{TP}\right)\right]$$

(4) $$\mathrm{TSI}\;\left(\mathrm{TN}\right)=10^{*}\;\left[5.453+1.694^{*}\;\ln\left(\mathrm{TN}\right)\right]$$

(5) $$\mathrm{TSI}\;\left(\mathrm{SD}\right)=10^{*}\;\left[5.118–1.94^{*}\;\ln\left(\mathrm{SD}\right)\right]$$

where TSI (Chl‐a), TSI (TN), TSI (TP) and TSI (SD) are the trophic state indices related to Chl‐a (mg/L), TN (mg/L), TP (mg/L) and SD (m), respectively. Five trophic levels were defined: oligotrophic (TSI < 30), mesotrophic (30 ≤ TSI ≤ 50), slightly eutrophic (50 < TSI ≤ 60), moderately eutrophic (60 < TSI ≤ 70) and highly eutrophic (TSI > 70) (Geng et al. 2022). Detailed information on the environmental variables of the trophic groups is provided in Dataset 1B.

### 2.3 River Sediment Microbial Cell Counts

Catalysed reporter deposition–fluorescence in situ hybridisation (CARD–FISH) was used to quantify river sediment microbial cell densities, following our previous studies (Li et al. 2023). The probe‐labelling peroxidases were EUB338 (bacteria, 5′‐GCW GCC WCC CGT AGG WGT‐3′) and Arch915 (archaea, 5′‐GTG CTC CCC CGC CAA TTC CT‐3′). Probe NON338 (5′‐ACT CCT ACG GGA GGC AGC‐3′) was used as a control. Cell enumeration was performed using ImageJ software. Each sample was analysed in triplicate, and the values were expressed as the means (*n* = 3).

### 2.4 16S rRNA Amplicon Sequencing

DNA was extracted from each sediment subsample using the E.Z.N.A. soil DNA kit (Omega Bio‐tek, Norcross, Georgia, USA), according to the manufacturer's instructions. The final DNA concentration was determined using a NanoDrop 2000 (Thermo Scientific, Waltham, USA), and DNA quality was checked using 1% agarose gel electrophoresis. High‐throughput sequencing was conducted by Majorbio Bio‐Pharm Technology Co. Ltd. (Shanghai, China). Bacterial/archaeal universal primers 515F and 806R, targeting the 16S rRNA gene V4 region, were used for PCR amplification as previously described (Li et al. 2020). PCR products were extracted from the 2% agarose gel and further purified using an AxyPrep DNA Gel Extraction Kit (Axygen Biosciences, USA) and quantified using Quanti Fluor‐ST (Promega, USA). Purified amplicons were pooled in equimolar amounts and paired‐end sequenced (2 × 300 bp) on an Illumina MiSeq platform (Illumina, San Diego, California, USA).

Raw FASTQ files were demultiplexed, quality‐filtered using Trimmomatic, and merged using FLASH. Operational taxonomic units (OTUs) were clustered with a 97% similarity cutoff using UPARSE, and chimeric sequences were identified and removed using UCHIME. To eliminate the effects of sampling intensity (number of reads) on the comparison of richness estimates, we randomly subsampled a fixed number of reads (*n* = 10,000) for each sample. Taxonomic assignments were performed using the SILVA 16S rRNA gene database (version 138). The distance‐based maximum likelihood method was used for phylogenetic analysis. Bootstrap analysis was performed with 1000 replications. MEGA software was used to construct phylogenetic trees. Alpha‐diversity metrics (observed OTU richness, Chao1 richness, Shannon and Simpson diversity indices and equitability index) were calculated using Mothur (Schloss et al. 2009).

### 2.5 Niche Breadth

To quantify habitat specialisation, we employed the niche breadth approach (Levins 1968) based on 16S amplicon sequencing. The formula is as follows:

(6) $$B_{j}=\frac{1}{\sum_{i=1}^{N}P_{\mathrm{ij}}^{2}}$$

where *B*_*j*_ is the habitat niche breadth of OTUj in a metacommunity, *P*_ij_ is the proportion of OTUj at site *i* and *N* is the total number of sites. A higher *B* value for the OTUs indicated a wider habitat niche breadth. In contrast, lower *B* values indicated that the OTUs occurred in fewer habitats and were unevenly distributed (Pandit et al. 2009). The average B‐value of all OTUs in a community was regarded as the community‐level B‐value (*Bcom*) (Jiao et al. 2020).

### 2.6 Neutral Community Model and Null Model

Neutral theory and niche theory can explain how species assemble into communities (Dini‐Andreote et al. 2015; Hubbell 2001). Neutral theory posits that stochastic processes, such as birth, death, speciation, immigration and limited dispersal, govern microbial communities. In contrast, niche theory argues that communities are shaped by deterministic biotic factors (such as species interactions, including competition and predation) and abiotic factors (like temperature and pH) (Ning et al. 2019; Zhou and Ning 2017). In this context, the neutral community model (NCM) (Sloan et al. 2006) and null model (Stegen et al. 2012) were employed to evaluate the drivers influencing community assembly processes.

NCM was used to determine the contribution of stochastic processes to the microbial community assembly by predicting the relationship between the frequency with which taxa occur in a set of local communities and their abundance across a wider metacommunity (Sloan et al. 2006). The model predicts that abundant taxa are more likely to be dispersed by chance and widespread across metacommunities, whereas rare taxa would be lost in different local communities owing to ecological drift. In this model, the estimated migration rate is a parameter for evaluating the probability that the random loss of an individual in a local community will be replaced by dispersal from the metacommunity; therefore, it is a measure of dispersal limitation. Higher *m* values indicate that the microbial communities are more dispersed. The formula used is as follows:

(7) $${\text{Freq}}_{i}=1-I\left(1/N\;|\;N^{*}m^{*}p_{i},N^{*}m^{*}\left(1-p_{i}\right)\right)$$

where Freq_*i*_ is the frequency of occurrence of taxon *i* across communities, *N* is the number of individuals per community, *m* is the estimated migration rate, *p*_*i*_ is the average relative abundance of taxon *i* across communities and *I*() is the probability density function of the beta distribution. *R* ^2^ indicates the fit of the parameter based on nonlinear least squares fitting (Jiao et al. 2020).

Nevertheless, the NCM did not account for all the variation in microbial communities, suggesting that other assembly mechanisms may also be at play, contributing to the observed nonneutral distribution (Zhou and Ning 2017; Liu et al. 2018). To explore this possibility, the null model was utilised to evaluate the relative roles of deterministic and stochastic processes. Null model analysis was performed using the framework described by Stegen et al. (2012) to classify community pairs according to the underlying drivers of species sorting (or selection), dispersal limitation, homogeneous dispersal and drift. The variation of both phylogenetic diversity and taxonomic diversity was measured using null model‐based phylogenetic and taxonomic *β*‐diversity metrics, namely *β*‐nearest taxon index (*β*NTI) and Bray–Curtis‐based Raup–Crick (RC_Bray_). A significant deviation (i.e., |*β*NTI| > 2) indicates the dominance of selection processes. *β*NTI < −2 indicates significantly less phylogenetic turnover than expected (i.e., homogeneous selection) while *β*NTI > +2 indicates significantly more phylogenetic turnover than expected (i.e., variable selection). Subsequently, RC_Bray_ was used to further partition the pairwise comparisons that were not assigned to selection (i.e., |*β*NTI| < 2). The relative influence of homogenising dispersal was quantified as the fraction of pairwise comparisons with |*β*NTI| < 2 and RC_Bray_ < −0.95. Dispersal limitation was quantified as the fraction of pairwise comparisons with |*β*NTI| < 2 and RC_Bray_ > 0.95. Fractions of all pairwise comparisons with |*β*NTI| < 2 and |RC_Bray_| < 0.95 were used to estimate the influence of ‘undominated’ assembly, which mostly consists of weak selection, weak dispersal, diversification and/or drift (Dini‐Andreote et al. 2015).

### 2.7 Metagenomic Sequencing and Functional Annotation

Metagenomic libraries were constructed using the Nextera DNA Library Preparation Kit (Illumina, USA). Sequencing was carried out on an Illumina HiSeq 3000 platform, generating 150 bp paired‐end reads. Raw reads were processed with Trimmomatic to remove barcodes and adapter sequences. After quality trimming, paired reads were merged and assembled into contigs using MEGAHIT. Protein‐coding genes were predicted from assembled contigs with Prodigal, and functional annotation was primarily performed against the Kyoto Encyclopaedia of Genes and Genomes (KEGG) database via GhostKOALA (Kanehisa et al. 2016). Additional annotations were conducted using eggNOG (Huerta‐Cepas et al. 2015), CARD (McArthur et al. 2013), ARDB (Liu and Pop 2009) and Resfams (Gibson et al. 2015). The relative abundance of antibiotic resistance genes (ARGs) was expressed as reads per million mapped reads (RPM).

### 2.8 Determination of Life‐History Traits

The estimation of microbial life‐history traits followed the procedure described by Chen et al. (Chen et al. 2021). The genome count for each sample was inferred from the mean sequencing coverage of the 35 single‐copy marker genes. The average 16S rRNA gene copy number was calculated as the ratio between the coverage of 16S rRNA genes and the estimated genome count, while the average genome size was obtained by dividing the total assembled base pairs by the genome count (Pereira‐Flores et al. 2019). To further characterise growth‐related traits, CUB within ribosomal protein‐encoding genes was assessed, and the minimum generation time (MGT, h^−1^) was inferred following the method proposed by Vieira‐Silva and Rocha (Vieira‐Silva and Rocha 2010). Ribosomal genes were identified by BLAST searches against a curated database of ribosomal proteins from reference genomes (Vieira‐Silva and Rocha 2010). For each ribosomal gene, CUB was quantified using the effective number of codons (ENC′) metric. The community‐level CUB was expressed as the inverse of the mean ENC′ value across all identified ribosomal genes (Novembre 2002). Based on CUB estimates, the MGT was predicted following the empirical relationship established by Vieira‐Silva and Rocha (Vieira‐Silva and Rocha 2010), and the potential maximum growth rate was defined as the inverse of the MGT (h^−1^) (Vieira‐Silva et al. 2016). Finally, the GC content of quality‐filtered reads was computed according to the protocol of Barberán et al. (Barberán et al. 2012).

### 2.9 Screening for Antibiotic‐Resistant Bacteria

The environmental occurrence of antibiotic resistance rates (ARRs) was assessed by calculating the resistance percentage, defined as the proportion of bacterial colonies capable of growth on antibiotic‐amended media relative to those grown on antibiotic‐free controls (Berendonk et al. 2015; Rizzo et al. 2013). The abundance of ARB in river surface sediments was quantified using the heterotrophic plate count (HPC) technique (Munir et al. 2011). Briefly, 0.1 g of each sediment sample was suspended in 10 mL of sterile 0.85% saline solution and serially diluted (10‐fold) for subsequent plating. A 100 μL aliquot of the diluted suspension was spread onto plate count agar (PCA) medium. Four media conditions were used: PCA supplemented with 50.4 μg mL^−1^ sulfamethoxazole, 16 μg mL^−1^ tetracycline, 15 μg mL^−1^ azithromycin and a control medium without antibiotics. Antibiotic concentrations followed the Clinical and Laboratory Standards Institute (CLSI, 2015) guidelines (Hsueh et al. 2010) and previous studies (Gao et al. 2012; Milaković et al. 2020). To inhibit fungal growth, all media were supplemented with 200 μg mL^−1^ cycloheximide. Plates were incubated at 37°C for 48–72 h, and colony‐forming units (CFUs) were enumerated to determine the proportion of resistant bacteria. All assays were performed in triplicate, and results were expressed as mean values (*n* = 3).

## 3 Results

### 3.1 Physicochemical Characteristics

**FIGURE 2:**
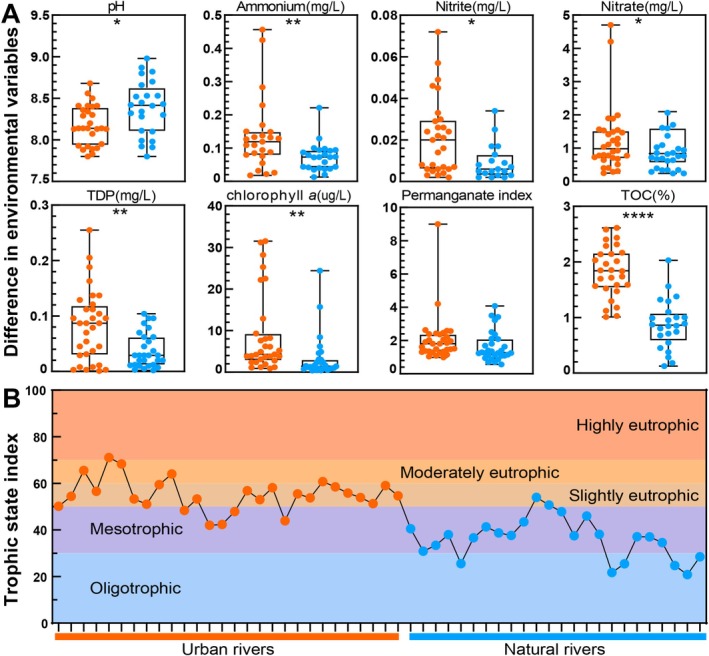
(A) Differences in environmental variables in river water (pH, ammonium, nitrite, nitrate, TDP, chlorophyll *a*, permanganate index) and sediment (TOC) samples between the UR (orange circles) and NR (blue circles). Asterisks denote significance levels (*p < 0.05; **p < 0.01; ****p < 0.0001). (B) Eutrophication assessment with the trophic state index (TSI). Five trophic levels were defined: oligotrophic (TSI < 30), mesotrophic (30 ≤ TSI ≤ 50), slightly eutrophic (50 < TSI ≤ 60), moderately eutrophic (60 < TSI ≤ 70) and highly eutrophic (TSI > 70) (Geng et al. 2022).

Sampling was conducted at 28 UR sites and 24 NR sites distributed along a distance gradient of 0.72–376 km (Figure 1). Measurements of river water revealed a broad range of physicochemical conditions, with pH values between 7.80 and 8.98, ammonium concentrations of 0.013–0.425 mg L^−1^, nitrite of 0.002–0.072 mg L^−1^, nitrate of 0.240–4.750 mg L^−1^, total dissolved phosphorus (TDP) of 0.001–0.255 mg L^−1^, chlorophyll *a* of 0.400–31.500 μg L^−1^ and permanganate index values of 0.560–9.000 (Figure 2A; Dataset 1A,B). Concentrations of ammonium, nitrite, nitrate, TDP, chlorophyll *a* and permanganate index were all significantly elevated in UR relative to NR (*p* < 0.05, Figure 2A). Likewise, TOC showed a pronounced enrichment in urban reaches, increasing from 0.127% to 2.029% in NR to 1.009%–2.612% in UR (*p* < 0.0001, Figure 2A; Dataset 2A).

The UR exhibited an average TSI of 53.07, ranging from 41.97 to 79.32 (Figure 2B, Dataset 1B). In contrast, the NR exhibited an average TSI of 36.23, ranging from 20.84 to 53.95. Collectively, these findings indicate that UR was predominantly eutrophic, while NR generally exhibited oligotrophic to mesotrophic conditions (see Methods for further details).

### 3.2 Microbial Community Abundance, Richness and Diversity

**FIGURE 3:**
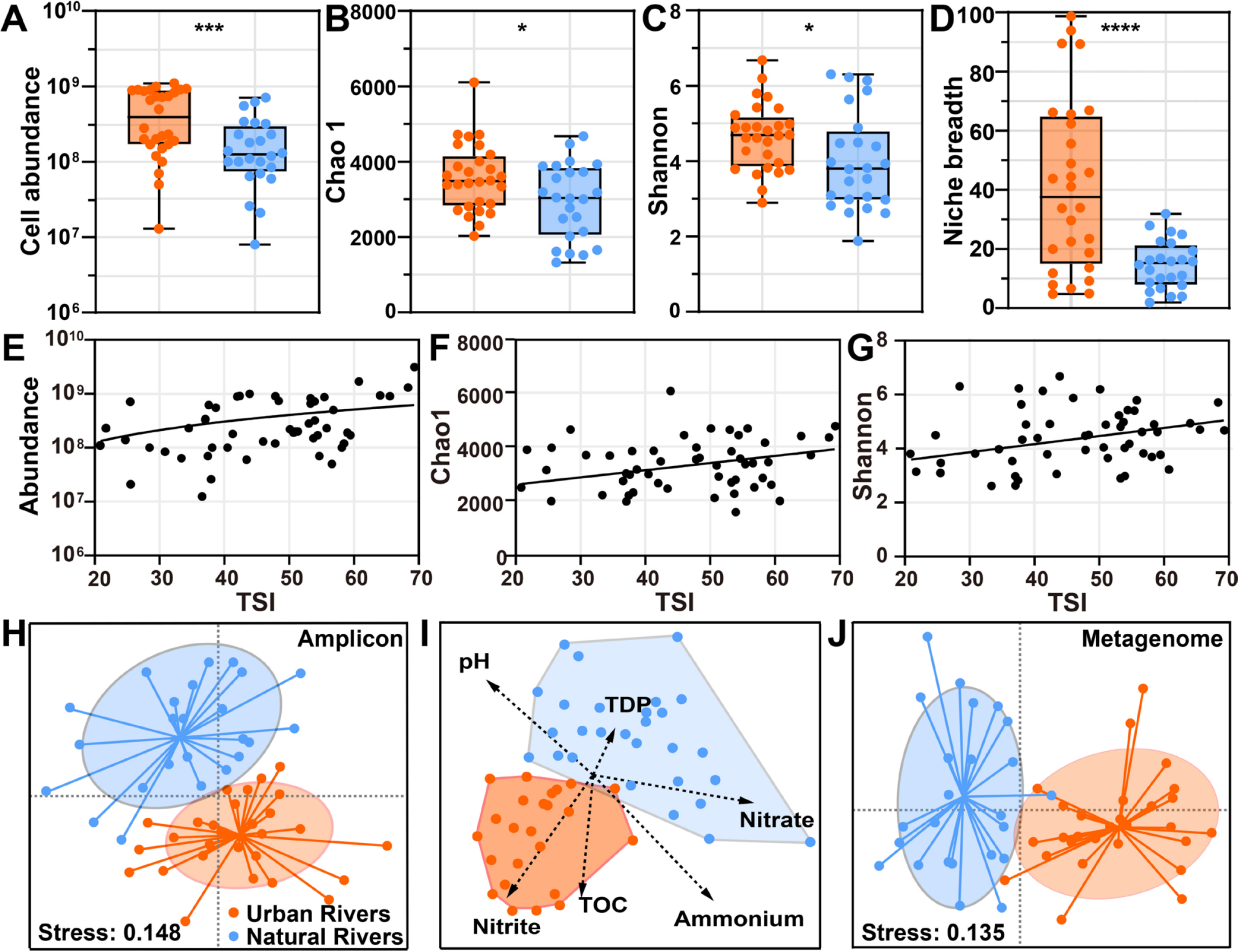
(A–D) Microbial abundance, Chao1 richness estimates, the Shannon index and niche breadth were investigated in the urban rivers (orange circles) and natural rivers (blue circles). (E–G) Pairwise relationships between the microbial abundance, Chao 1 richness, Shannon index and TSI for observed communities. (H and J) Similarity of microbial community composition (amplicon sequencing) and function (metagenomic sequencing) visualised by NMDS between the urban rivers and natural rivers. (F) db‐RDA illustrating community similarity constrained by environmental variables, represented as arrows whose lengths indicate the strength of correlations with RDA axes.

The microbial abundance was 1.3 × 10^7^–1.1 × 10^9^ cells g^−1^ and 8.0 × 10^6^–7.1 × 10^8^ cells g^−1^ in the UR and NR sediment samples, respectively (Figure 3A, Dataset 2A). Microbial abundance was significantly higher in UR than in NR (*p* < 0.001, Figure 3A), and populations tended to increase with rising TSI (Figure 3E), suggesting that human‐induced eutrophication enhances riverine microbial biomass.

Using universal primers for bacteria and archaea, 3,309,566 high‐quality 16S rDNA gene sequences were generated (Dataset 2A), and these sequences were clustered into 18,038 OTUs at a 97% similarity level (Dataset 2B). A range of 2030–6104 chao1 OTUs and 2.89–6.68 Shannon index were observed in the UR, and a range of 1321–4678 chao1 OTUs and 1.88–6.31 Shannon indices were observed in the NR (Figure 3B,C). Both Chao1 richness and Shannon diversity were significantly higher in UR than in NR (*p* < 0.05). Moreover, niche breadth (Figure 3D) and phylogenetic distance (Figure 5A) were also greater in UR than NR microbial communities.

**FIGURE 4:**
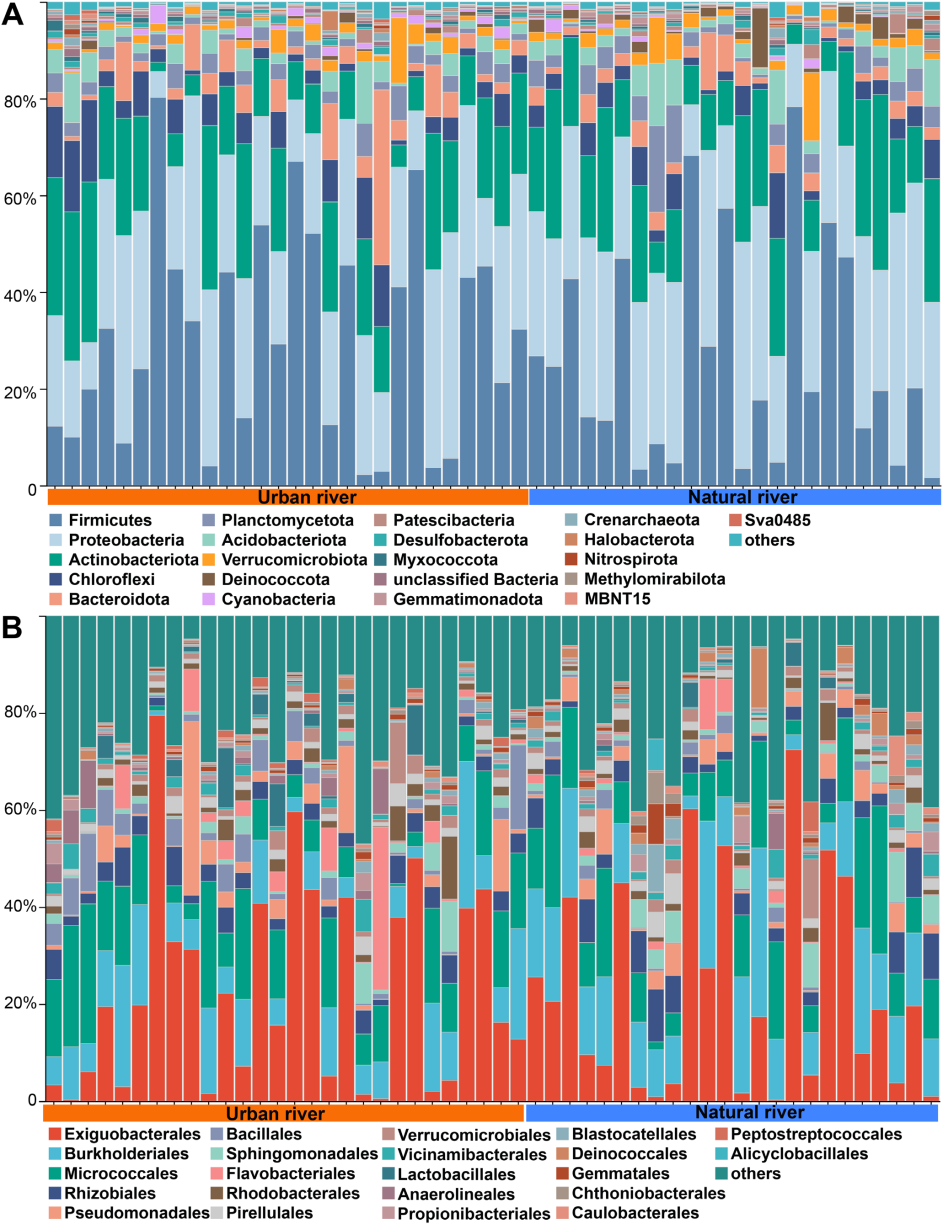
Community composition showing the top abundance of phyla (A) and orders (B) in UR and NR based on amplicon data.

We further profiled prokaryotic diversity across taxonomic levels from phylum to species (Dataset 2B–H). UR harboured 74 phyla and 3599 species, whereas NR contained 80 phyla and 3736 species. In UR, the dominant phyla were Firmicutes (30.40%), Proteobacteria (26.06%), Actinobacteriota (17.64%), Chloroflexi (5.89%), Bacteroidota (5.34%), Acidobacteriota (3.19%), Planctomycetota (3.01%), Verrucomicrobiota (2.21%), Cyanobacteria (0.92%) and Desulfobacterota (0.68%) (Figure 4A, Dataset 2C). In NR, the most abundant phyla were Proteobacteria (31.74%), Firmicutes (25.89%), Actinobacteriota (17.96%), Planctomycetota (4.08%), Chloroflexi (3.94%), Acidobacteriota (3.88%), Bacteroidota (3.52%), Verrucomicrobiota (2.69%), Deinococcota (1.69%) and Patescibacteria (0.82%).

At the order level, the dominant groups in UR were Exiguobacterales (22.91%), Micrococcales (12.30%), Burkholderiales (10.59%), Pseudomonadales (4.00%), Bacillales (3.95%), Rhizobiales (3.81%), Flavobacteriales (2.99%), Rhodobacterales (2.25%), Lactobacillales (1.89%) and Sphingomonadales (1.85%). In NR, dominant taxa included Exiguobacterales (2.73%), Burkholderiales (15.13%), Micrococcales (12.55%), Rhizobiales (4.88%), Pseudomonadales (3.18%), Sphingomonadales (2.96%), Pirellulales (1.99%), Rhodobacterales (1.75%), Deinococcales (1.66%) and Verrucomicrobiales (1.64%) (Figure 4B, Dataset 2E).

At the OTU level, nonmetric multidimensional scaling (NMDS) showed that the UR harboured significantly dissimilar microbial communities from the NR (Figure 3H; PERMANOVA pseudo F = 2.1455, *p*.adj = 0.024, 999 permutations; ANOSIM *p* = 0.03). Distance‐based redundancy analysis (db‐RDA) revealed that nitrogen had a significant impact on shifts within the microbial community of UR (Figure 3I).

### 3.3 Microbial Community Assembly Processes

**FIGURE 5:**
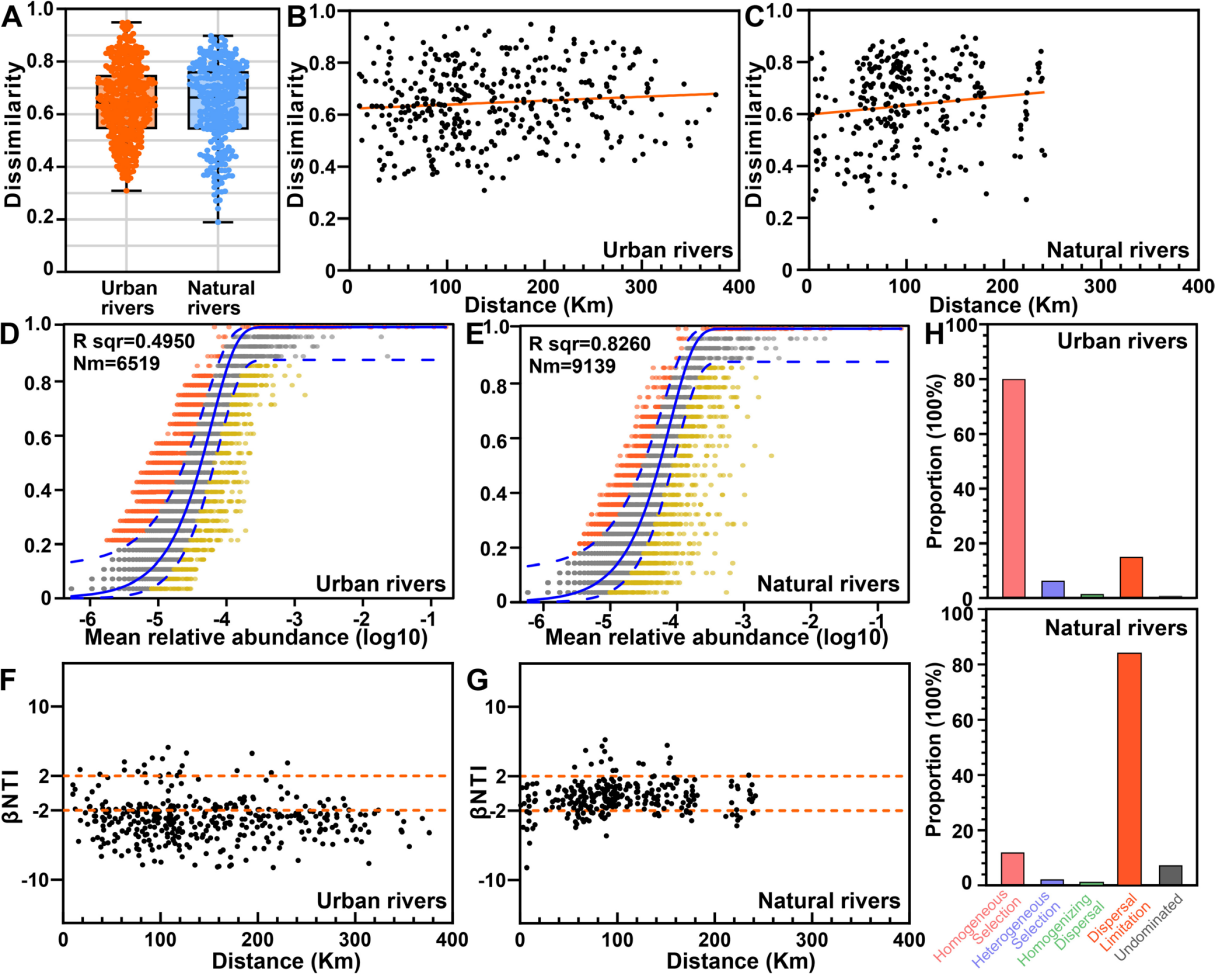
(A) Compositional differences between the urban rivers and natural rivers were assessed using the Bray–Curtis distance. The box plot illustrates the community dissimilarities observed between the two regions. (B and C) Distance‐decay curves depicting the relationship between Bray–Curtis similarity and geographical distances among sampling sites. (D and E) The fit of the NCM for community assembly is presented, for the urban rivers and natural rivers. (F–H) Variations in microbial communities explained by stochastic processes (such as dispersal limitation) and deterministic processes (such as homogeneous or heterogeneous selection) based on null model analysis.

Across the ~400 km river network, NR exhibited higher β‐diversity (Figure 5A) and a more pronounced distance–decay relationship (DDR) (Figure 5B,C, Dataset 2I), indicating that turnover rates in community composition differed between UR and NR. To investigate the factors influencing community assembly, the NCM and null model were used to assess the relative influences of stochastic and deterministic processes.

The NCM showed the relationship between the occurrence frequency of OTUs and relative abundances (Figure 5D,E), suggesting that stochastic processes contribute to microbial community assembly in both UR and NR. However, as eutrophication increased, the influence of stochastic processes decreased, accounting for 82.6% of the community variance in NR and only 49.5% in UR (Figure 5D,E). The *Nm* value was also higher in NR (*Nm* = 9139) than in UR (*Nm* = 6519), indicating more extensive species dispersal among microorganisms in NR compared with UR.

Null model analyses indicated that intensified eutrophication corresponded with a reduced contribution of stochasticity and an enhanced role of homogeneous selection in UR (Figure 5F–H). This pattern implies that eutrophication functions as a potent environmental filter, strengthening deterministic assembly mechanisms while diminishing random community dynamics within UR microbiomes.

### 3.4 Microbial Life‐History Strategies and Functional Profiles

**FIGURE 6:**
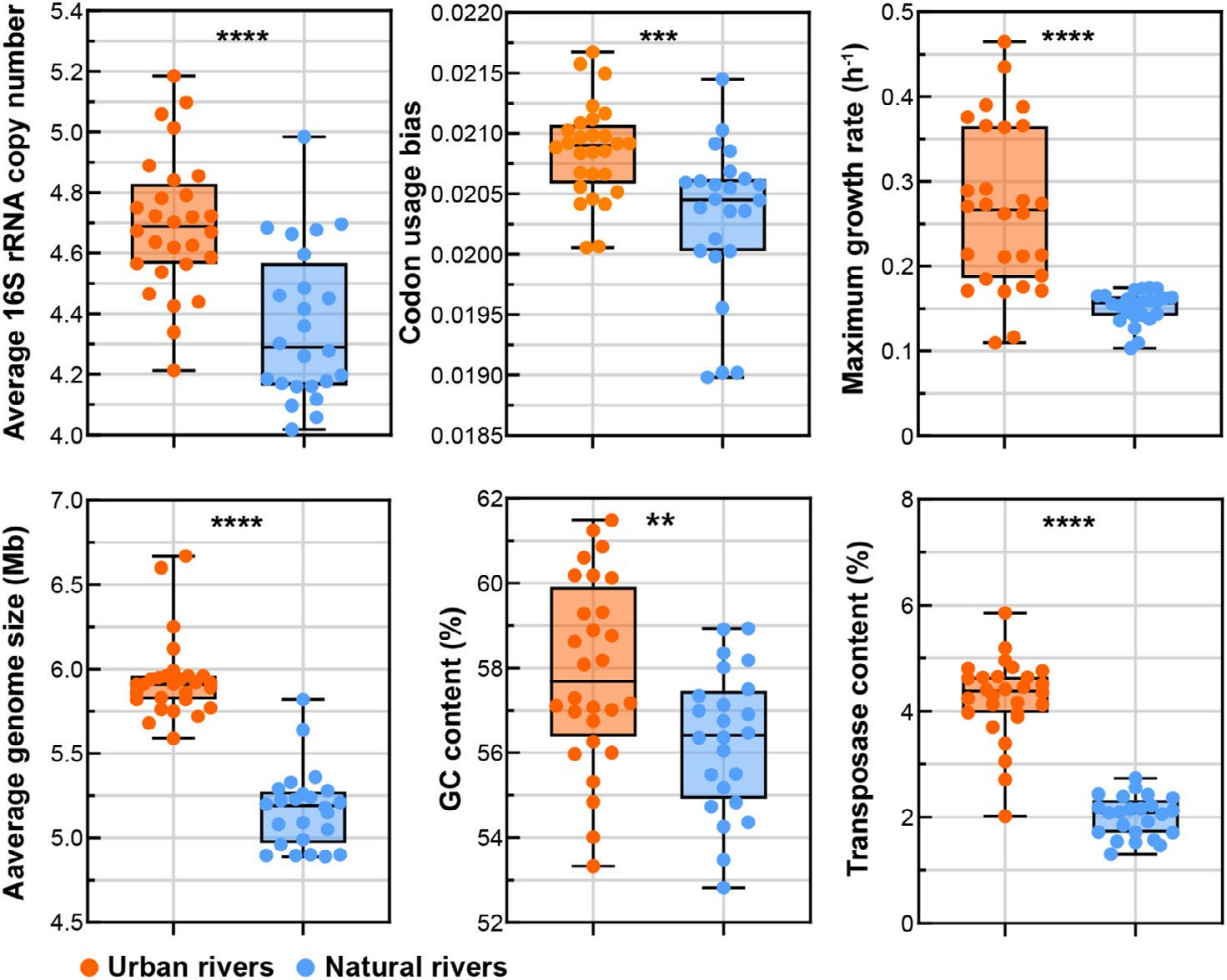
Microbial life‐history traits. Box plots show median values for UR versus NR: 16S rRNA copy number (4.69 vs. 4.28), CUB (0.0209 vs. 0.0204), maximum growth rate (0.2664 vs. 0.1567 h^−1^), genome size (5.91 vs. 5.19 Mb), GC content (57.68% vs. 56.41%) and transposase content (4.37% vs. 2.98%). Significance was evaluated using the Wilcoxon rank‐sum test with FDR correction (Benjamini–Hochberg). Asterisks denote significance levels (**p < 0.01; ***p < 0.001; ****p < 0.0001).

We performed metagenomic sequencing on 52 sediment samples, generating 1034.07 Gb of raw data (Dataset 3A). From the metagenomic data, we calculated key microbial life‐history traits to compare microbial strategies between UR and NR (Figure 6). The median 16S rRNA gene copy number was notably higher in UR (4.69 vs. 4.28), reflecting enhanced ribosomal content and growth capacity. CUB, a measure of translation efficiency, was also greater in UR (0.0209 vs. 0.0204). Correspondingly, the predicted maximum growth rate was higher in UR (0.2664 h^−1^ vs. 0.1567 h^−1^), supporting a copiotrophic strategy. Genomic analysis showed that UR microbiomes had larger genomes (5.91 Mb vs. 5.19 Mb) and higher GC content (57.68% vs. 56.41%). Additionally, UR samples had a higher proportion of transposase genes (4.37% vs. 2.98%).

**FIGURE 7:**
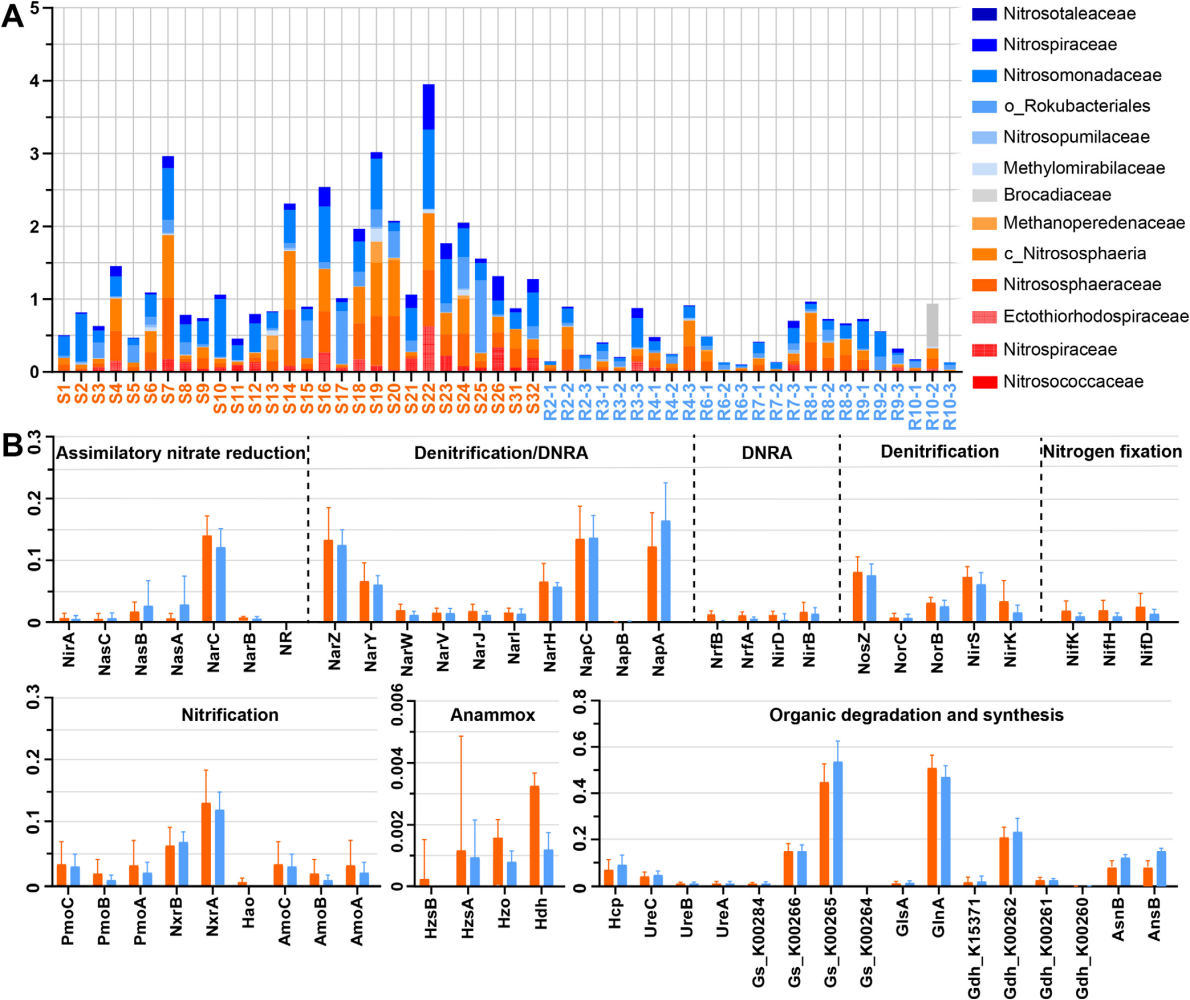
(A) Relative abundance of microbial families involved in nitrogen cycling, inferred from amplicon sequencing data. (B) Relative abundance of sequence reads encoding dissimilatory and assimilatory nitrogen‐cycling proteins, derived from metagenomic data and expressed as proportions of total nitrogen‐cycling pathways. Orange bars represent urban river samples, and blue bars represent natural river samples.

When comparing the annotated KEGG functions of the UR and NR, we found that the similarity in metagenomic functional genes (Figure 3J, Dataset 3B) mirrored the patterns observed in the 16S rRNA gene amplicons (Figure 3H, Dataset 2B). This indicates a strong correlation between taxonomic and functional profiles, with a Pearson correlation coefficient of 0.92. Given that nitrogen cycling was a fundamental component of the microbiomes in all samples (Figure 7A), we focused on the distribution of nitrogen metabolism (Figure 7B, Dataset 3D). We identified 58 gene families that encode nitrogen‐cycling proteins associated with processes including nitrification, denitrification, dissimilatory nitrate reduction to ammonia (DNRA), anammox, ammonia assimilation, nitrogen fixation and organic nitrogen mineralisation, as identified using the NCycDB database (Tu et al. 2018) for both UR and NR (Dataset 3D).

Among the nitrification processes, ammonia oxidation (amo) and nitrite oxidation (nxr) were dominant in UR (Figure 7B). Furthermore, genes associated with the reduction of nitrate to nitrite (nar), as well as those related to nitrite reduction during DNRA (nrf) and denitrification (nir) pathways, showed higher abundance in UR (Figure 7B). In addition to encoding complete nitrification–denitrification pathways, genes related to anammox were also significantly more prevalent in UR (*p* < 0.05). In comparison, NR showed a notably higher prevalence of nasA genes linked to the assimilatory nitrate reduction pathway. This increase was also observed in genes involved in amino acid synthesis, such as asparagine synthetase (asnB) and L‐asparaginase II (ansB) (Figure 7B).

### 3.5 Antibiotic Resistome

**FIGURE 8:**
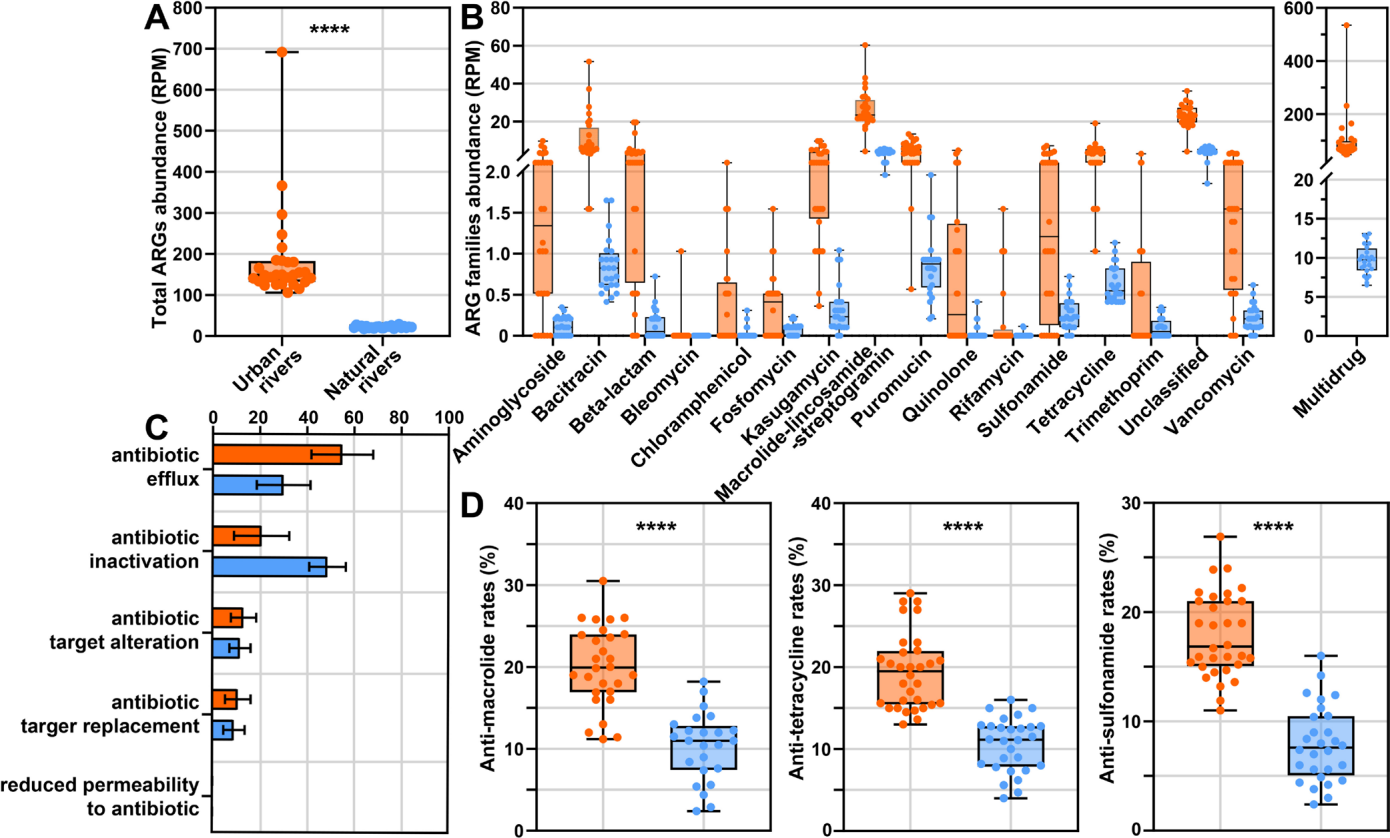
(A and B) Relative abundance of total ARGs and ARG families in UR and NR. ARG read counts were normalised to sequencing depth, expressed as reads per million (RPM). (C) Dominant antibiotic resistance mechanisms identified in UR and NR. (D) ARRs test. Orange circles represent UR samples, and blue circles represent NR samples.

Metagenomic datasets were mapped against the ResFams database (Dataset 3E), which revealed 18 ARG families, including those conferring resistance to aminoglycosides, bacitracins, bleomycins, β‐lactams, chloramphenicols, fosmidomycins, fosfomycins, kasugamycins, macrolide‐lincosamide‐streptogramins, puromycins, polymyxins, quinolones, rifamycins, sulfonamides, trimethoprims, tetracyclines and vancomycins (Dataset 3E). The relative abundance of ARG sequences was markedly higher in UR compared with NR (*p* < 0.0001, Figure 8A,B; Dataset 3E). Moreover, transposase genes, used as indicators of horizontal gene transfer potential, were considerably more abundant in UR (4.24%) than in NR (2.01%) (Figure 6; Dataset 3G).

To validate the metagenomic evidence, ARRs were further determined for three commonly applied antibiotic classes: macrolides, tetracyclines and sulfonamides (Figure 8D). In UR, ARRs ranged from 11.2% to 30.5% for azithromycin, 13.0% to 29.0% for tetracycline and 11.0% to 26.9% for sulfonamide, whereas NR samples exhibited lower values (2.318.2%–18.2%, 4.0%–16.0% and 2.4%–16.0%, respectively). Overall, ARRs were consistently higher in UR than in NR (Figure 8D), corroborating the metagenomic results that eutrophic UR harbour an enhanced potential for antibiotic resistance dissemination.

## 4 Discussion

### 4.1 Eutrophication Shifts Riverine Microbial Diversity and Community Composition

Our analyses showed pronounced differences in microbial abundance, richness, diversity and community composition between UR and NR (Figures 3 and 4). These differences were accompanied by clear patterns of niche differentiation (Figure 3D), which remained evident even after the exclusion of rare biosphere taxa (< 0.1% of total sequences). db‐RDA further indicated that nitrogen was a major environmental driver shaping the UR microbiomes (Figure 3I). These findings demonstrate that anthropogenic activities, particularly those that alter water quality, strongly affect both the α‐ and β‐diversity of riverine microbial assemblages.

According to the productivity‐resource hypothesis (Ibarbalz et al. 2019), greater nutrient availability and primary production can sustain larger and more diverse microbial populations. UR, receiving continuous inputs of organic carbon and nutrients from urban runoff and agricultural discharge, exhibited markedly greater primary productivity (Figure 2A). In addition, the organic carbon in UR ecosystems is typically labile and enriched in carbohydrates and amino acids, which stimulate microbial respiration, growth and biomass accumulation (Geng et al. 2022). Consequently, the nutrient‐enriched UR provided favourable conditions that enhanced microbial abundance and diversity relative to NR.

Eutrophication was also accompanied by taxonomic shifts, as evidenced by the significant enrichment of Bacteroidota (*p* < 0.05), Firmicutes (*p* < 0.05) and Spirochaetota (*p* < 0.001) in UR. Both Bacteroidota and Firmicutes are dominant taxa of the human gut microbiome (Wang et al. 2018; Ley et al. 2006; Chen et al. 2019), whereas Spirochaetota are known to include potential pathogens linked to various human diseases (Chen et al. 2019). The higher abundances of Bacteroidota, Firmicutes and Spirochaetota in UR suggest contamination by untreated domestic wastewater originating from residential areas. In contrast, Planctomycetota, a typical riverine lineage known for its sensitivity to heavy metals and inorganic nitrogen (Xu et al. 2023; Fuerst and Sagulenko 2011), identified as the seventh most dominant phylum overall but showed a marked decline in UR sediments (Dataset 2C). This reduction likely reflects metal and nutrient pollution in UR systems.

Moreover, amplicon data revealed enrichment of taxa associated with nitrogen transformations in UR (Figure 7A), and metagenomic profiles revealed that ammonia‐oxidising bacteria (AOB) and archaea (AOA), which catalyse the oxidation of ammonia to nitrate through the enzymes ammonia monooxygenase (Amo) and hydroxylamine oxidoreductase (Hao), were particularly abundant in UR (Figure 7B). This result indicates that anthropogenic nitrogen enrichment substantially enhances nitrification capacity in eutrophic river sediments. In parallel, the increased prevalence of genes involved in DNRA, denitrification and anammox further suggests intensified microbial nitrogen turnover and removal potential (Figure 7B). Nevertheless, these nitrogen transformation pathways may concurrently release reactive intermediates such as nitrite, as well as potent greenhouse gases including nitric oxide and nitrous oxide, thereby threatening riverine ecosystem stability.

A more direct linkage between eutrophication and microbial communities was reflected in the profiles of ARGs (Figure 8A–C, Dataset 3E) and ARRs (Figure 8D). Both ARG and ARR levels were markedly higher in UR than in NR (*p* < 0.0001 and *p* < 0.001, respectively; Figure 8A–D). Likewise, the relative abundance of transposase genes was significantly higher in UR (*p* < 0.0001). Transposases can mobilise ARGs by inserting them into plasmids and other mobile genetic elements, thereby facilitating interspecies transfer through conjugation. Such plasmid‐mediated exchanges accelerate the spread of antibiotic resistance within microbial communities, contributing to the formation of environmental reservoirs of multidrug‐resistant bacteria. The potential for these mobile ARGs to disseminate to human or animal pathogens, either directly in aquatic habitats or indirectly via food webs, poses a growing ecological and public health concern for riverine ecosystems.

### 4.2 Eutrophication Reshapes Riverine Community Assembly Processes and Life‐History Strategies

UR and NR exhibited distinct taxonomic and functional characteristics, which could be attributed to environmental differences (Figures 2 and 3). However, identifying the mechanisms governing microbial community assembly is challenging because of the complex feedbacks between biotic interactions and abiotic gradients that act across spatial and temporal scales (Martiny et al. 2011). To disentangle these effects, we employed the NCM and null model analyses to evaluate the relative importance of stochastic and deterministic processes.

The NCM indicated that stochastic processes explained 49.5% of the variation in UR and 82.6% in NR (Figure 5D,E), implying a stronger stochastic influence in NR. Moreover, null model results showed that deterministic selection, particularly homogeneous selection, dominated in UR (Figure 5F–H). The NCM and null model analyses suggest that eutrophication exerts strong environmental filtering, thereby enhancing deterministic assembly and constraining stochasticity. Conversely, the weaker physicochemical gradients in NR allowed greater immigration, ecological drift and random colonisation, thereby amplifying stochastic influences.

To further examine microbial adaptation to eutrophication, we compared key life‐history traits derived from metagenomic data. Microbial communities in UR exhibited significantly higher 16S rRNA gene copy numbers (Figure 6), indicating increased ribosomal content and protein synthesis potential, features typical of copiotrophic taxa thriving in nutrient‐enriched habitats (Ling et al. 2022; Nemergut et al. 2015; Kearns and Shade 2018). Ribosomal genes in UR communities also showed stronger CUB (Figure 6), reflecting selection for enhanced translational efficiency, a hallmark of fast‐growing microorganisms (Vieira‐Silva and Rocha 2010). Consistently, predicted growth rates were also higher in UR (Figure 6), confirming the dominance of *r*‐strategists in eutrophic UR.

Genomic features also reflected microbial adaptation to local nutrient conditions. The mean guanine–cytosine (GC) content was markedly higher in UR microbial genomes (Figure 6). Biosynthetically, each GC base pair incorporates eight nitrogen atoms, whereas adenine–thymine (AT) pairs contain seven (Okie et al. 2020). Based on the ‘resource‐driven selection theory’ (Wang et al. 2024), nitrogen scarcity tends to select for genomes with lower GC proportions (Chuckran et al. 2023). Consequently, the nitrogen enrichment characteristic of UR (Figure 2A) may favour GC‐rich genomes, underscoring the role of eutrophication in driving microbial genomic adaptation and evolutionary trajectories.

We also found that microbial communities in UR possessed significantly larger genomes than those in NR (Figure 6). This observation aligns with the concept that organisms in nutrient‐poor environments undergo genome streamlining (Lauro et al. 2009; Lever et al. 2015). The Black Queen Hypothesis (Morris et al. 2012) suggests that genomic reduction can be adaptive by minimising biosynthetic costs and fostering cooperative interactions through the exchange of public metabolic goods. Consequently, microbes inhabiting NR tend to maintain compact, energy‐efficient genomes containing only core functional genes required for survival. In contrast, continuous inflows of organic matter and nutrients, including nitrogen and phosphorus, from human activities create resource‐rich environments (Figure 2). These conditions selectively favour *r*‐strategists with large, versatile genomes encoding diverse metabolic pathways. Expanded genome content enhances metabolic flexibility and broadens ecological niches (Figure 3D), enabling microbial populations to exploit diverse substrates, alleviate direct competition and promote phylogenetic diversification (Figure 5A).

Overall, the trade‐off between growth and survival represents a fundamental principle in microbial ecology. However, the widespread presence of slow‐growing oligotrophic microorganisms in various ecosystems indicates that *r*‐strategists are not always favoured (Zhu and Dai 2024). Numerous studies have indicated that although *r*‐strategists gain short‐term benefits under nutrient‐rich conditions, *K*‐strategists generally exhibit greater persistence and resistance to environmental stress (Beulig et al. 2022; Jørgensen and Boetius 2007; Morono et al. 2020; Wörmer et al. 2019; Jørgensen and Marshall 2016). As a result, while eutrophication stimulates rapid microbial proliferation, it may erode community resilience by favouring opportunistic taxa. Therefore, the predominance of deterministic processes observed in our study has important management implications, suggesting that nutrient regulation could effectively steer community assembly. This insight offers a foundation for ecological restoration, as reducing eutrophic pressure, for instance, by controlling nutrient inputs from human activities, may curb the dominance of r‐strategists and foster more resilient, K‐strategist‐oriented microbial communities.

## 5 Conclusion

Our study demonstrates that anthropogenic eutrophication profoundly reshapes riverine microbial diversity, functional attributes and life‐history strategies. Elevated nutrient levels in urban waters stimulate the dominance of opportunistic, fast‐growing *r*‐strategists; however, this restructuring may reduce the long‐term stability and environmental buffering capacity of microbial communities. Thus, nutrient‐induced selective pressures not only reconfigure microbial life‐history strategies but also weaken the ecological integrity and resilience of river ecosystems.

## Author Contributions

**Haizhou Li:** conceptualization, investigation, funding acquisition, writing – original draft, writing – review and editing, visualization, validation, methodology, software, formal analysis, project administration, resources, supervision, data curation. **Jing Fu:** investigation, software, validation. **Xiangyu Fan:** investigation, visualization. **Zhiwei He:** investigation, visualization. **Yuekai Wang:** investigation, software, validation. **Shanshan Yang:** investigation, validation, software. **Jiawang Wu:** investigation, validation, software. **Li Wu:** investigation, validation, software. **Jin Zhou:** funding acquisition, investigation, supervision.

## Conflicts of Interest

The authors declare no conflicts of interest.

## Supporting information


**Dataset: 1.** Geographic coordinates and geochemical characteristics of sampling sites.

**Dataset: 2.** 16S rRNA gene amplicon sequencing results of microbial communities.

**Dataset: 3.** Metagenomic sequencing results of microbial communities.

## Data Availability

The data that support the findings of this study are openly available in the National Genomics Data Centre at (PRJCA018972). Metagenomic sequencing data are available upon request.
